# Scalpels and Standards: What Students Consider Ethical, Unethical, and Ethically Challenging in the Anatomy Lab

**DOI:** 10.1007/s40670-025-02565-8

**Published:** 2025-11-13

**Authors:** Homaira M. Azim, So Hyun Jeon, Christian J. Faller, Vivian P. Bui, Evan W. Fairweather, Mekha M. Varghese, Dimitrios E. Bakatsias, Michael N. Cardiges

**Affiliations:** 1 Department of Neurobiology and Anatomy, Drexel University College of Medicine, Philadelphia, PA USA; 2 Lewis Katz School of Medicine at Temple University, Philadelphia, PA USA; 3 Temple/St. Luke’s School of Medicine, Bethlehem, PA USA; 4 Drexel University College of Medicine, Philadelphia, PA USA

**Keywords:** Cadaveric dissection, Anatomy education, Medical education, Professional identity formation, Medical professionalism, Ethical conduct, Medical ethics

## Abstract

**Background:**

Human dissection in medical education presents an ethically complex terrain shaped by institutional norms, peer culture, and personal values. While formal codes and donor protections have evolved, little is known about how students themselves define and interpret ethical conduct in the gross anatomy lab. This qualitative study explored medical students’ perceptions of ethical behavior during dissection.

**Methods:**

Twenty students from all four years of medical school at a single U.S. institution participated in one-on-one, semi-structured interviews. Transcripts were analyzed inductively using open coding to identify recurring themes. Credibility was enhanced through analyst triangulation, reflexive dialogue, and use of illustrative quotes.

**Results:**

Three overarching themes emerged: (1) Ethical practices were linked to faculty role-modeling, peer and self-monitoring, and mindfulness during dissection; (2) Unethical practices included inappropriate jokes, careless handling of donor bodies, and unprofessional behavior; and (3) Ethically challenging situations involved uncertainty about donor consent, tension between objectification and personification of the body, and unspoken norms discouraging emotional expression. These dynamics often went unaddressed by formal instruction or policy, leaving students to navigate them independently.

**Conclusions:**

Students engage with dissection not only as a technical task but also as a moral experience. While institutional rules and professional standards are necessary, they are often insufficient on their own to help students smoothly navigate the ethical and emotional complexities of the anatomy lab. Anatomy education should incorporate structured opportunities for ethical and emotional reflection, reinforce transparency around body donation, and foster a culture that validates emotional responses. These practices may better support professional identity formation and align ethical conduct with students’ lived experiences.

## Background

Ethical conduct in the anatomy lab has long been influenced by broader societal, religious, and cultural values, shaping how medical students are taught to approach the traditionally sensitive practice of human dissection [1, 2]. Ethical codes, typically rooted in principles of rights, fairness, societal benefit, or specific virtues, have historically defined the standards for behavior and professional practice in medical education [3, 4]. Although anatomical dissection for scientific purposes dates back to third century BCE Alexandria, the practice was considered a violation of sacred norms surrounding bodily integrity and funeral rites for much of its history [1, 5, 6]. While evolving ethical frameworks have alleviated many of these tensions in the past century, the act of human dissection remains controversial in various cultural and religious contexts [7]. Furthermore, there is no universally accepted ethical code for human dissection; significant variation persists in how different societies regulate and interpret the practice [8]. These historical and cultural complexities underscore the need for continued ethical scrutiny and institutional guidance wherever dissection is undertaken.

To maintain public trust and uphold donor dignity, modern anatomical education now adheres to rigorous ethical standards [9, 10]. Donated bodies must be acquired through voluntary, informed consent, and used exclusively for educational purposes, with any form of commercial exploitation strictly prohibited [11, 12]. Written consent must detail the procedures to be performed, the duration of body retention, and the final disposition of remains [8, 11]. These practices mark a significant ethical shift from earlier centuries, particularly from the late 19th to the late 20th century, during which U.S. medical institutions disproportionately relied on unclaimed bodies—often those of the poor, institutionalized, or marginalized groups—for dissection [13]. This history raises deep concerns about structural injustice and dignity. Today, further safeguards such as individualized storage of body donors, restricted lab access, prohibition of photography, and stringent handling protocols are common [14, 15]. While these measures aim to ensure respectful conduct, consistent enforcement remains a logistical challenge [16, 17].

Despite strict ethical frameworks, medical students continue to experience a range of emotional and ethical reactions during dissection. Reported approval rates of cadaveric dissection vary widely—from 19.66% among Indian medical students to 79% among female Saudi students—highlighting the influence of cultural and individual factors [18–21]. Many students report initial feelings of anxiety (50%) and fear (28.2%) during their first exposure to the lab, navigating these reactions through diverse coping mechanisms [18, 22, 23]. These include objectification (viewing the donor as a specimen) to promote detachment, and personification (engaging in humanizing reflection) to maintain empathy [24, 25]. In addition to managing these emotions, students may also face ethical challenges surrounding lab conduct. While most students identify disrespectful behaviors—such as joking or photographing cadavers—as unethical, there is often tension between their personal moral discomfort and the perceived educational necessity of dissection [23,25,]. Awareness of the history of unethical procurement practices also plays a role in shaping contemporary student ethics, strengthening their appreciation for informed consent and the dignity of voluntary body donation [13].

Building on this historical and cultural foundation, contemporary research has increasingly turned to the lived experiences of students themselves. Recognizing the emotional and ethical complexity of cadaveric dissection, medical schools have implemented a range of interventions aimed at reinforcing professionalism, empathy, and reflective practice. Since gross anatomy is one of the first clinical experiences for medical students, it is often seen as a formative setting for professional identity development and moral growth [25]. Structured opportunities for emotional processing—such as peer discussions, written reflections, and group-based activities—help students develop empathy and moral sensitivity while also normalizing emotional responses to death and dissection [26–29]. Other strategies include peer and self-evaluations, which promote ethical accountability and reflection [30], and clear behavioral standards, such as prohibiting inappropriate jokes or disrespectful language, to reinforce the mutual respect central to physician-patient relationships [31]. Humanizing the donor has increasingly emerged as a powerful perspective used to deepen students’ ethical awareness. These efforts—such as referring to donors as “first patients,” integrating donor biographies into the curriculum, holding memorial ceremonies, and placing respectful signage in the lab—aim to cultivate compassion, altruism, and a stronger sense of responsibility [29, 32]. Additionally, elective courses that reinforce anatomy through spaced repetition and longitudinal integration—such as the elective described by Azim et al. [33]—have been shown to enhance student confidence and create sustained engagement with anatomical learning, which may in turn support deeper reflection on professional values over time. By fostering emotional connection between students and donors and ethical reflection, such interventions position the dissection lab not only as a space for anatomical learning, but as a unique moral training ground for future physicians.

While institutional efforts to enforce ethical conduct are well-documented [15, 34, 35], there remains a significant gap in understanding how students themselves define and interpret ethical behavior in the dissection lab. Emerging evidence suggests that students’ ethical reasoning may, in fact, diverge considerably from institutional expectations based on individual identity, background, and moral frameworks. Nawras et al. [36] found considerable variability in student responses to ethically charged scenarios, with factors such as age and gender influencing ethical sensitivity. While some students expressed strong discomfort with certain behaviors (e.g., photography of cadavers), others showed little concern, even when institutional policies had been clearly communicated. These findings challenge the efficacy of the one-size-fits-all ethical mandates and highlight the importance of aligning institutional expectations with students’ internalized value systems [37].

Given the anatomy lab’s role as a student’s first sustained encounter with ethical ambiguity in medical education, it is essential to examine how students themselves conceptualize ethical conduct. Do their personal codes of conduct align with formal institutional standards? How do they navigate morally complex situations that may not be explicitly addressed by policy? Despite the critical role of anatomy education in shaping professional identity, few studies have addressed these questions directly. The present study seeks to address this gap by exploring how medical students define, interpret, and navigate ethical behavior during dissection. Assessing the degree of alignment between personal values and institutional expectations can inform the development of more nuanced, student-centered approaches to ethics education in medical curricula.

## Methods

### Study Setting and Participants

This study was conducted as part of a larger research initiative aiming to describe medical students’ experiences with anatomy education as it pertains to ethics, morality, and professional identity formation. All enrolled students, encompassing all four years of undergraduate medical education (M1–M4) at a single U.S. medical school were invited to participate. At the time of data collection, there were approximately 1,400 medical students enrolled across nine campuses of the medical school. Recruitment was conducted via institutional email addresses. Two email invitations were sent 15 days apart, and after the second message, 20 students volunteered to participate. This number was deemed sufficient for the scope of the study, aligning with qualitative research standards that emphasize depth and richness of data over numerical generalizability. Students were offered the choice of a free lunch or a $10 Amazon gift card, as an incentive for their participation.

Participant characteristics are summarized in Table 1. A detailed description of the study participants is included to highlight the sample’s diversity, address potential concerns about representation [38], and support the transferability of findings to comparable educational contexts [39].

**Table 1:** Study participant characteristics

Participant Characteristics	*n*
Total Participants	20
***Class Year***	
First-Year (M1)	8
Second-Year (M2)	3
Third-Year (M3)	5
Fourth-Year (M4)	4
***Gender***	
Men	12
Women	8
***Race/Ethnicity***	
Caucasian	12
South Asian	2
African American	2
Multiracial	4

### Data Collection

A total of 20 one-on-one interviews were conducted in person, each lasting approximately one hour. All interviews were conducted by the same person (HA), who was an associate instructor at the time of data collection and had no role in grading students. Moreover, all participants had already completed the anatomy course at the time of interview, ensuring that there was no possibility of coercion or concern about course evaluation. Transcription was completed using Zoom’s automated service. This process produced approximately 20 h of interview data and over 1,000 pages of raw text. While students had completed anatomy at different campuses and in different years, this variation enriched the dataset by capturing diverse perspectives on lab cultures, faculty role-modeling, and dissection environments. The interview guide included broad, open-ended questions designed to elicit students’ reflections on their anatomy lab experiences (see Supplementary File 1 for the full interview guide).

### Data Analysis

All data were anonymized and assigned unique participant identifiers prior to analysis to ensure participant confidentiality. Participants were labeled numerically (1–20) along with their year of study. For example, “P9-M4” refers to Participant 9, a fourth-year medical student. Interview transcripts were analyzed using an inductive, open coding approach. An initial review of transcripts helped identify emerging themes, which were then refined through iterative, in-depth analysis to allow patterns and meanings to naturally emerge from the data.

To strengthen interpretive reliability, analyst triangulation was employed in this study, a strategy that enhances rigor and credibility by minimizing individual bias. Three members of the research team (HA, SJ, and VB) independently reviewed and coded the transcripts using an inductive, open coding approach. Initial codes were developed individually and then compared in group discussions to reconcile differences and reach consensus on key themes and subthemes. These thematic structures were subsequently reviewed and endorsed by all members of the research team. Thematic saturation was reached by approximately the 16th–18th interview, at which point few new concepts were emerging. Additional strategies included reflexive dialogue among the research team to elucidate and examine assumptions, and the use of illustrative verbatim quotes to substantiate interpretations with direct evidence from participants’ narratives.

The study was based on a constructivist research paradigm, which emphasizes the co-creation of meaning between researchers and participants [40, 41]. Rather than applying a predefined theoretical framework, the analysis was guided by constructivist principles, focusing on participants’ subjective experiences and the context-dependent nature of knowledge. Our constructivist orientation was also reflected in the design of the interview guide, which included broad, open-ended questions inviting participants to reflect on their experiences in the anatomy lab, their judgments about appropriate or inappropriate conduct, and the factors that shaped those judgments. Rather than testing predefined categories, the questions were intended to elicit participants’ own meaning-making processes around ethical issues in dissection. In analysis, we further aligned with this orientation by attending to how students constructed and negotiated meaning through their reflections on institutional norms, peer culture, and personal values.

## Results

**Fig. 1:**
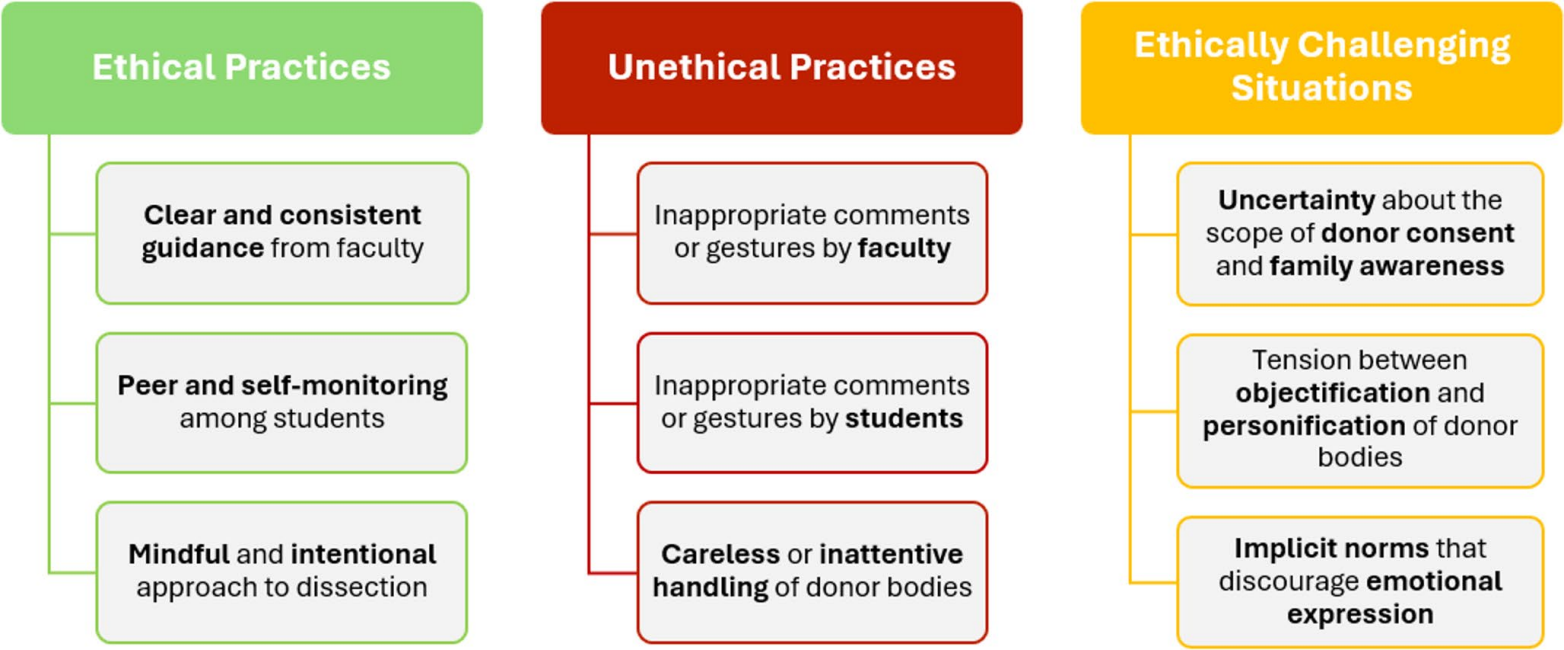
Student Perceptions of Ethical Conduct in the Gross Anatomy Lab

Our study revealed three overarching themes describing medical students’ perceptions of ethical conduct in the anatomy lab: (1) ethical practices, (2) unethical practices, and (3) ethically challenging situations (see Fig. 1). Ethical practices were linked to institutional and peer reinforcement of respectful behavior and mindful engagement during dissection. Unethical practices included inappropriate comments or actions by students and faculty, as well as careless treatment of donor bodies. Ethically challenging situations reflected emotional, cultural, or cognitive tensions, such as uncertainty about donor consent, balancing detachment and empathy, and implicit norms discouraging emotional expression.

Across all three themes, students highlighted recurring dynamics involving faculty roles, individual attitudes, and peer-to-peer interactions. These dimensions appeared in ethically positive, negative, and ambiguous forms, underscoring that ethical conduct in the lab is shaped by a complex interplay of individual actions, group norms, and institutional context.

This figure illustrates students’ perceptions of ethical and unethical conduct in the gross anatomy lab, highlighting key behaviors, institutional influences, and interpersonal dynamics as described during interviews.

1. **Ethical practices**.

Students identified three primary components that contributed to ethical conduct in the anatomy lab: clear and consistent guidance from institutional leaders, a culture of peer and self-monitoring, and mindfulness during dissection.

a **Clear and consistent guidance provided by faculty**

Participants emphasized the importance of explicit rules for ethical conduct being introduced and reinforced throughout the course. They felt that these guidelines should not just be posted as a document on the course learning management system, but actively and meaningfully discussed by faculty members. Students appreciated the faculty who took the time to do this and understood this gesture as a demonstration of good and ethical conduct.

*Some instructors were good at instilling the sense of physical respect… I remember a few of the guys from my class who were making jokes about her*,* our cadaver… Dr. X pulled us aside afterwards… it was pretty upsetting*,* and I was really happy that Dr. X heard it and knew that it wasn’t okay and pulled us aside to discuss it. So*,* I was really impressed by his response to that.* [P15-M1]

*Some of the rules in the anatomy lab… like little pieces of body tissue would go to the same bin because it all needed to be cremated with the same body… it kind of reminds you that this is a person*,* and it was like showing respect to them. Like… we are saving your remains so you can get a proper remembrance. I thought that it was very ethical of the anatomy faculty to really stress that hey*,* I don’t care how small that bit is*,* it doesn’t go in the trash. It goes with the rest of this person because it is a person.* [P1-M2]

b **A Student culture of peer and self-monitoring a student culture of peer and self-monitoring**

Many students described informal mechanisms of accountability within dissection groups, where peers actively maintained ethical standards and corrected one another when necessary.

*I think every time I heard something that kind of indicated too much objectification of the body*,* I think I did a decent job of correcting that by jumping in and commenting back. And I think everyone*,* at least the people in my pod*,* we were all like*,* if someone got out of the line*,* we were quick to get involved and kind of remind each other. So*,* I think there was a certain level of self-correction that we all went by.* [P17-M1]

*If someone did something*,* then you will hear it*,* and nobody wants to mess up the first semester of med school ever. They were just like in their best behavior. The professor and the TAs were roaming around at all times… And again*,* we were just so close to each other*,* so no one wants to be the person who did something stupid*,* you know*,* that everyone knows. So*,* I think that helped.* [P6-M3]

c **Mindfulness and intentionality during dissection**

Participants highlighted the importance of treating each dissection act with purpose and care, often drawing on personal, cultural, or philosophical values to guide their approach.

*I was very careful; I treated it like it [my dissection] was an art. Like*,* you know*,* it wasn’t just like here we go rip his face off… I wanted to do it really nicely and leave everything intact.* [P8-M1]

*In Buddhism there is a principle of mindfulness*,* which is basically like not to waste your action or thoughts*,* and make sure you mean everything you say and do as much as possible. I think that definitely came to the body that I was using… I would say to myself if that was one of my loved ones*,* the thing I want to happen is to make sure every single cut was mindful… that every cut was purposeful. Like*,* you really have to mean that cut and need to make it*,* not just to tear things apart.* [P14-M1]

2 **Unethical practices**

This theme includes behaviors or attitudes that were considered disrespectful or inappropriate by students. Participants described instances involving faculty, peers, or themselves, where ethical expectations were not upheld.

a **Inappropriate comments or gestures made by faculty**

Some students shared experiences where faculty members engaged in behaviors they viewed as unprofessional or disrespectful, which they felt undermined the gravity of the human dissection experience.

*We had a professor who put coins in the [donor’s] eyes. I thought it was kind of disrespectful… It was kind of an odd gesture. I think that was kind of the only sign of disrespect that I saw*,* I think overall people were respectful. [P7-M2]*

*And then another thing was that Dr. Y*,* like she once grabbed a scalpel and stabbed it in the lady’s [donor’s] butt. So*,* I thought like that was not respectful. You can’t just stab anywhere… [P16-M1]*.

b **Inappropriate comments or gestures made by students**

Although infrequent, participants recalled student remarks or jokes they found troubling. While often overlooked or left unaddressed, these comments contributed to a lingering sense of discomfort among some students.

*Most of my classmates were pretty professional*,* but sometimes*,* some comments were made*,* you know*,* comments that weren’t worth making. They didn’t help anyone. They didn’t contribute anything… Others kind of ignored the comment or certainly didn’t feed into it. Like some people would make jokes like*,* oh*,* do the handshakes*,* and like… I was like*,* look*,* I wouldn’t do anything that I’d perceive this person wouldn’t want me to do.* [P3-M4]

*I remember that one table in the anatomy lab found a perforated nasal septum*,* and someone yelled across the anatomy lab that*,* ‘oh my gosh*,* they must have been doing cocaine!’ And our professor was like*,* we can’t say that; that’s inappropriate.* [P13-M4]

*And then*,* there was one of my table mates*,* he called our cadaver “babe” or something*,* and I was very uncomfortable with that. I guess he didn’t really mean anything bad*,* and he is kind of a goofy person*,* so… anyways*,* that was something that I remember.* [P16-M1]

c **Careless or inattentive handling of the bodies**

Students also described moments when donor bodies were handled in ways that seemed careless or thoughtless, reflecting a lack of appropriate respect or awareness.

*Mostly people were respective*,* but there were also definitely people who would just let an arm drop and not take the time to set it down gently*,* and were kind of rough with it*,* not caring about it… It’s a matter of curtesy*,* I don’t know… So*,* if that was my body and a med student was throwing my legs around*,* I would not be happy about it. I think the donors envision a medical student who would take care of them*,* or you wouldn’t give your body away if you knew that someone was gonna cut you up and mess around with you*,* you know.* [P4-M3]

*I only liked to have the part of the body exposed that I was working on*,* but some people had just their entire body*,* like face and everything uncovered. I don’t think that’s ok. But it’s just like everyone’s perception.* [P5-M1]

3. **Challenging ethical situations**

This theme captures emotionally and morally complex situations that did not neatly align with conventional understandings of ethical or unethical behavior, but nonetheless provoked both internal conflict and collective dilemmas.

a **Uncertainty about the details of donor consent and family awareness**

Students expressed concern and curiosity about the process of body donation and whether donors and their families were fully aware of the details involved in anatomical dissection.

*I was thinking of that*,* these people have families*,* are their families okay with this? I kept thinking that what if it was my sister with all of this happening to her… so what if their families didn’t want this? [P5-M1]*

*I am not entirely sure what goes into people’s minds when they donate their body… I don’t know that process… like how much specificity one gets when they donate their body to science… it makes me wonder if some people would still agree to be a donor if they knew that at some point we will be using a regular saw from Home Depot to transect their pelvis*,* you know. And it might not be even necessary to give them that kind of gross details*,* but… [P11-M2]*.

b **Balancing objectification and personification**

Students grappled with viewing the donor body simultaneously as an anatomical specimen and as a once-living person. Navigating this tension was often challenging and prompted internal conflict, as well as reflection on their personal ethical values.

*I think everybody*,* including me*,* said something at some point that if the patient could hear or their family*,* they would not be happy. Because we would look at it objectively*,* and like*,* ‘oh my patient is thin*,* thank God!’ And if you had an overweight patient then all you could comment about is like ‘oh my God*,* they are so fat*,* how did they live like that’… so things like that. It’s just objectifying the patients to the point that*,* that’s the thing*,* if you desensitize yourself too much*,* then it stops you from seeing that if they were your patient*,* and if the patient or their family were here*,* could you still say what you said. And if not*,* then don’t say it.* [P6-M3]

*Sometimes*,* like you objectify the donor to an extent that you completely forget that this was a person and needs to be treated with the due respect. And other times some students fall completely on the side of personalizing their cadaver that all they see is a person who is a human*,* is somebody’d loved one*,* has had a life*,* etc. In that situation too*,* they can’t really function and learn and the entire process kind of goes for nothing.* [P14-M1]

c **Implicit norms discouraging emotional expression**

Many students perceived unspoken expectations that displaying emotion about donor bodies or the dissection process would be viewed by peers as a sign of weakness. This perception influenced how they processed their experiences and engaged with others in the lab.

*I feel like there wasn’t that much space for discussing these emotions among med students and I felt like even wanting to discuss the emotional aspects of the dissection was in itself a sign of weakness. Which I feel is reflected in the medical profession in general. It was disappointing to me that the school has been offering health and wellness resources*,* but the culture is with us*,* even us the first-year medical students*,* if we are already making those standards*,* then there is less hope for how a future can be*,* and how physicians are gonna be emotional and vulnerable and express pain and stuff like that.* [P17-M1]

*One donor actually recorded a video prior to her death and that was played for all of us on the first day of anatomy… It would almost be like weird or would have been perceived as odd for someone to walkout. So*,* if I was the one who felt uncomfortable*,* I wouldn’t want to walk out*,* because I would be afraid of the perception of my classmates*,* like what would they think of me*,* like*,* am I not strong enough to see this*,* am I weak?* [P13-M4]

*Before going to lab here*,* I was really anxious*,* I couldn’t sleep the night before*,* I was really scared to touch them or cut them or anything like that. And that was when… I felt like there was this culture among students that*,* like you have to wanna use the scalpel a lot*,* and you can never be grossed out or nervous*,* and every part of body is the same to cut through. Like*,* I mean this is not what people said directly*,* but it’s like if you acted that way then you cannot be a surgeon. And the students have to act that way*,* if you want to be at the top of your class then you have to act that way.* [P9-M4]

## Discussion

This study, conducted at a single U.S. medical school, explored medical students’ perceptions of ethical conduct in the gross anatomy lab, revealing a spectrum of responses: ethical practices were valued, unethical behaviors were often silently condemned, and ethically challenging situations—despite their frequency—typically went unaddressed by faculty or formal guidelines. This observation is drawn directly from participants’ reflections within the Unethical Practices theme, where several students described instances of inappropriate comments or behaviors that were ignored or overlooked by both peers and instructors. These accounts suggested that silence or inaction, rather than overt approval, often contributed to the normalization of certain unprofessional behaviors. Taken together, the three themes—ethical practices, unethical practices, and ethically challenging situations—collectively reflect the moral complexity of dissection, illustrating how students’ ethical reasoning was influenced by the interplay of institutional norms, peer dynamics, and emotional responses during this formative stage of professional identity development.

In this context, “institutional norms” refer not to formal school-wide policies, but to the implicit expectations, shared practices, and cultural patterns that shaped the ethical tone of the anatomy lab. Students often described these norms as an unspoken understanding of “how things are done” rather than rules formally codified in curricular or professional guidelines. This distinction is important, as it underscores the influence of informal culture on students’ developing sense of professionalism and ethical conduct.

Amid this moral complexity, students emphasized the importance of clear and consistent guidance from faculty, peer and self-monitoring, and mindfulness during dissection. These elements helped foster a respectful and intentional learning environment. This aligns with prior calls for anatomy instruction to emphasize professionalism and ethical behavior [16, 28, 32, 42]. Notably, many students did not passively conform to institutional norms—they described active efforts to maintain peer accountability and correct inappropriate behavior when it arose. Such actions reflected an emerging sense of agency and ethical responsibility, suggesting that students were not merely shaped by the lab’s culture but also contributed to reshaping it. These intentional approaches, drawn from cultural and personal values such as Buddhist mindfulness, added depth to their ethical engagement in the lab.

While the terms ethics and morality are often used interchangeably, they carry distinct meanings [43–45]. Ethics generally refers to principles that guide individual or institutional conduct based on concepts such as justice, virtue, or benefit to society [3, 4], while morality often describes the values or expectations of a particular community (Stanford Encyclopedia). This distinction was reflected in our participants’ reflections: some students framed their conduct using moral language rooted in personal, cultural, or spiritual beliefs—such as respect for the donor’s body as sacred—whereas others emphasized adherence to professional expectations and codes of behavior modeled by faculty and peers. While overlapping, this distinction may explain students’ differing responses to the same dissection experiences, as those guided primarily by moral values sometimes expressed discomfort or reverence, while others grounded in professional ethics tended to approach dissection more pragmatically. Professionalism in medicine requires engagement with both ethical and moral domains [46–48], and anatomy education is a key space where professional identity formation begins [28, 42, 49]. This process—through which students internalize expected behaviors and reconcile them with personal values—can be supported or disrupted by experiences in the lab [50–52].

Many participants expressed uncertainty about the body donation process, particularly whether donors and their families were fully informed of what anatomical dissection for educational purposes entails. This concern emerged as part of the Ethically Challenging Situations theme, where students described feelings of unease stemming from limited information about how donations occur and what happens to the bodies after dissection. Several participants noted that they had received only brief or procedural explanations before entering the lab, which left them uncertain about the transparency of the process. This theme was noted repeatedly across transcripts and confirmed independently by three analysts. These concerns echo findings from previous studies that highlight gaps in student understanding of the consent process and discomfort with its perceived opacity [20,36. Although formal procedures are in place to safeguard donor consent, a lack of transparency in how these processes are communicated to students can contribute to ethical discomfort. Incorporating open discussions of consent, donor intent, and institutional responsibility into anatomy education could help address these concerns and reinforce trust in the ethical foundations of the course.

A central ethical tension described by participants involved balancing objectification and personification of the donor body. Students described a push and pull between detachment as a learning strategy and efforts to remain emotionally grounded. This finding supports prior research showing that students often oscillate between viewing the donor as a “learning tool” and as a person deserving of dignity and remembrance [22, 24, 25]. Moreover, longitudinal studies have shown that objectification may increase over time [25], suggesting a risk of emotional numbing [53]. While some detachment may be protective, excessive disengagement can erode empathy. Including strategies such as donor memorials, letters to donor families, and sharing donor biographies in anatomy courses may help maintain donor personhood and reinforce humanistic values [29].

Among the more striking findings involved unspoken community norms that discouraged emotional expression among medical students. Many students described anxiety about appearing “weak” or overly sensitive in front of their peers. Even when institutional wellness resources were available, students felt constrained by a culture of emotional stoicism. These dynamics align with broader critiques of the hidden curriculum—the informal transmission of values through institutional culture—that may contradict formal instruction in empathy and reflection [54–57]. Students perceived emotional control as necessary to appear competent, echoing past critiques of gendered and performative norms in medical training [58, 59]. This dynamic may hinder healthy emotional development and compromise long-term well-being. Medical educators aiming to promote professionalism must address not only formal content but also the implicit values that shape how students interpret and respond to ethically complex situations related to anatomy dissection lab experience.

In addition to shaping professional identity formation, ethical tensions in the anatomy lab may also influence the quality of the learning environment and students’ academic engagement. Prior research has shown that emotional discomfort, unaddressed ethical ambiguity, and hidden curriculum dynamics can impede concentration, limit reflective learning, and diminish confidence in skill acquisition [25, 54, 55]. Conversely, supportive faculty role-modeling and structured opportunities for ethical reflection have been associated with stronger learning outcomes and deeper engagement in anatomy education [16, 28]. Recognizing this interplay highlights the importance of fostering an environment where ethical concerns are not only acknowledged but actively integrated into pedagogical strategies.

In light of these findings, we recommend that anatomy education intervene beyond the passive enforcement of behavioral norms—such as simply posting ethical guidelines online—and instead create more space for ethical and emotional reflection. Structured interventions such as peer-led debriefings, reflective writing assignments, guided small-group discussions, or engagement with donor or family narratives may help students process the emotional weight of human dissection, confront hidden norms, and integrate personal values into their emerging professional identities.

While this study offers valuable insights, certain limitations should be considered. It was conducted at a single U.S. medical school, which may limit the transferability of findings. Although the final sample size of 20 participants was appropriate for the scope of this qualitative study and sufficient to reach thematic saturation, larger or more in-depth studies could further probe these issues and enhance the transferability of findings. Future research with broader samples across institutions may help confirm and extend these insights. While our sample included diversity across class year, gender, race/ethnicity, and campus sites, it cannot capture the full sociocultural range of medical student experiences. In qualitative research, the goal is to generate in-depth insight rather than statistical generalizability, yet we acknowledge that additional perspectives could further enrich understanding. Future studies with larger, multi-institutional, and more socioculturally stratified samples would be valuable to confirm and extend the themes identified here and to enhance transferability of findings. Additionally, participants may have underreported ethically problematic behaviors due to social desirability bias. Given the sensitivity of discussing disrespectful or unprofessional conduct in the anatomy lab, students may have been reluctant to disclose certain details or may have framed their experiences in ways that reflected more positively on themselves or their peers. This possibility should be considered when interpreting the relative frequency or severity of unethical behaviors described.

Nevertheless, this study makes a meaningful contribution to the growing literature on ethics and professional identity formation in anatomy education. By centering student voices, it highlights how learners interpret dissection through the lenses of institutional norms, peer culture, and personal values. Understanding these dynamics is essential not only for shaping anatomy curricula that teach the structural intricacies of the human body, but also for cultivating the professionalism, empathy, and moral awareness needed in future physicians. While many of the issues raised may be familiar to experienced anatomy educators, such perspectives are rarely published in research literature. By systematically documenting students’ voices, this study draws broader attention from the medical education community and underscores the need for sustained action to address persistent ethical challenges in the anatomy lab.

## Conclusions

This study reveals the ethical landscape of the gross anatomy lab as experienced by medical students — highlighting a dynamic interplay among institutional guidance, peer norms, and personal values. While many students emphasized the importance of faculty role modeling, clear rules, and mutual accountability, they also described witnessing or participating in behaviors that deviated from these ideals. Notably, ethically challenging situations such as uncertainty about the scope of donor consent, tensions between objectification and personification, and unspoken norms that discouraged emotional expression were often left unaddressed by formal policies or instruction. These findings suggest that anatomy education must extend beyond rule enforcement to actively engage with the emotional and ethical complexities of human dissection. Offering structured opportunities for student reflection, transparency around the body donation process, and a learning environment that allows for emotional vulnerability may help bridge the gap between institutional expectations and students’ lived experiences. As the anatomy lab often represents students’ first authorized act of physical intervention on a human body, it serves as a formative space for developing professionalism, empathy, and respect. Ensuring these values are intentionally cultivated—not passively assumed—can support students’ moral development and shape the kind of physicians they ultimately become.

## Data Availability

Majority of data generated and analyzed during this study are included in this published article. Complete raw datasets generated in this study are available from the corresponding author on reasonable request.
